# Effect of Age on the Association Between Waist-to-Height Ratio and Incidence of Cardiovascular Disease: The Suita Study

**DOI:** 10.2188/jea.JE20130004

**Published:** 2013-09-05

**Authors:** Yukako Tatsumi, Makoto Watanabe, Yoshihiro Kokubo, Kunihiro Nishimura, Aya Higashiyama, Tomonori Okamura, Akira Okayama, Yoshihiro Miyamoto

**Affiliations:** 1 Department of Preventive Cardiology, National Cerebral and Cardiovascular Center, Suita, Osaka, Japan; 1 国立循環器病研究センター; 2 Department of Mathematical Health Science, Graduate School of Medicine, Osaka University, Suita, Osaka, Japan; 2 大阪大学大学院医学系研究科 総合ヘルスプロモーション科学; 3 Department of Environmental and Preventive Medicine, Hyogo College of Medicine, Nishinomiya, Hyogo, Japan; 3 兵庫医科大学 環境予防医学; 4 Department of Preventive Medicine and Public Health, Keio University, Tokyo, Japan; 4 慶應義塾大学医学部 衛生学公衆衛生学; 5 The First Institute for Health Promotion and Health Care, Japan Anti-tuberculosis Association, Tokyo, Japan; 5 公益財団法人結核予防会 第一健康相談所総合健診センター

**Keywords:** waist-to-height ratio, age difference, cardiovascular disease

## Abstract

**Background:**

Waist-to-height ratio (WHtR) has been shown to be a useful screening tool for metabolic syndrome and cardiovascular disease (CVD). We investigated the association of WHtR with CVD incidence by age group.

**Methods:**

We conducted a 13.0-year cohort study of Japanese adults (2600 men and 2888 women) with no history of CVD. WHtR was calculated as waist circumference (cm) (WC) divided by height (cm). We stratified participants by sex and age group (30–49, 50–69, ≥70 years). Using the Cox proportional hazards model, we calculated hazard ratios (HRs) and 95% CIs for CVD in relation to WHtR quartile for participants aged 50 to 69 years and 70 years or older.

**Results:**

Men aged 50 to 69 years in the highest quartile had significantly increased risks of CVD and coronary heart disease as compared with the lowest quartile; the HRs (95% CI) were 1.82 (1.13–2.92) and 2.42 (1.15–5.12), respectively. Women aged 50 to 69 years in the highest quartile had a significantly increased risk of stroke (HR, 2.43; 95% CI, 1.01–5.85). No significant results were observed in men or women aged 70 years or older. The likelihood ratio test showed that the predictive value of WHtR was greater than that of WC among men aged 50 to 69 years.

**Conclusions:**

The association between WHtR and CVD risk differed among age groups. WHtR was useful in identifying middle-aged Japanese at higher risk of CVD and was a better predictor than WC of CVD, especially in men.

## Abstract (ja)

**背景：:**

腹囲だけでなく、腹囲・身長比も循環器疾患およびメタボリックシンドロームのスクリーニングに有用であることが報告されているが、年齢の影響を考慮した報告は限られている。そこで、本研究では腹囲・身長比と循環器疾患の発症の関連を年齢階級別に検討した。

**方法：:**

1989-1994年に大阪府吹田市住民から無作為抽出された循環器疾患（冠動脈疾患・脳卒中）の既往のない30-83歳の5488名（女性2888名、男性2600名）を平均13年間追跡した。性別・年齢階級別(50-69歳、70歳以上)に腹囲・身長比の四分位と循環器疾患発症との関連を、Cox比例ハザードモデルを用いて検討した。

**結果：:**

50-69歳男性において、総循環器疾患および冠動脈疾患で有意にリスクが上昇し、第4四分位のハザード比(95%信頼区間)は、第1四分位を基準として、それぞれ1.82（1.13-2.92）、2.42 (1.15-5.12)であった。女性では脳卒中で有意にリスクが上昇し、ハザード比は2.43 (1.01-5.85)であった。男女とも、70歳以上では有意なリスクの上昇は見られなかった。尤度比検定より、腹囲・身長比は50-69歳の男性において腹囲より高い予測力を示した。

**結論：:**

腹囲・身長比と循環器疾患発症の関係は年齢階級により異なっていた。腹囲・身長比は日本人の中年男女において循環器疾患発症のハイリスク者を特定するのに有用であり、特に中年男性においては、腹囲よりも予測力が高かった。

## INTRODUCTION

Obesity and central obesity are closely tied to metabolic risks.^1^^,^^2^ Waist circumference (WC) is an index of central obesity^3^ and is an important component in the diagnostic criteria for metabolic syndrome.^4^ Several meta-analyses have reported an association of WC with cardiovascular disease (CVD) and mortality.^5^^,^^6^ Recently, waist-to-height ratio (WHtR) was shown to be a useful global clinical screening tool for cardiometabolic risk and CVD.^7^^,^^8^

WHtR is easy to measure, and the cut-off point for WHtR is subject to less ethnic variation.^7^^,^^8^ However, WHtR could differ among age groups because whole-body fat distribution and WC change considerably with age^9^^,^^10^ and because height differs among generations.^11^ It is thus important to consider age in assessing the association between WHtR and CVD risk, but few previous studies have done so.^12^^,^^13^ Therefore, in this long-term prospective cohort study of a Japanese urban population, we investigated the effect of WHtR on CVD risk among participants classified by age group.

## METHODS

### Study population

The Suita Study is a prospective population-based cohort study of an urban area of Japan and was established in 1989. The details of this study have been described elsewhere.^14^^–^^16^ Briefly, 6407 men and women aged 30 to 83 years underwent a baseline survey at the National Cerebral and Cardiovascular Center between September 1989 and March 1994. Among them, a total of 919 were excluded due to past history of CVD (*n* = 208), loss to follow-up (*n* = 535), and missing data (*n* = 176). The remaining 5488 participants (2600 men and 2888 women) were included in the analysis. This cohort study was approved by the Institutional Review Board of the National Cerebral and Cardiovascular Center.

### Baseline examination

Blood samples were centrifuged immediately after collection, and a routine blood examination was performed, including measurement of serum levels of total cholesterol and glucose. About 96% of participants had fasted for at least 8 hours before the blood test. Well-trained physicians used a standard mercury sphygmomanometer to measure blood pressure in triplicate on the right arm after 5 minutes of rest. Hypertension was defined as systolic blood pressure of at least 140 mm Hg, diastolic blood pressure of at least 90 mm Hg, or use of antihypertensive agents. Diabetes was defined as a fasting plasma glucose level of at least 7.0 mmol/L (126 mg/dL), a non-fasting plasma glucose level of at least 11.1 mmol/L (200 mg/dL), or use of antidiabetic agents. Hypercholesterolemia was defined as a total cholesterol level of at least 5.7 mmol/L (220 mg/dL) or use of antihyperlipidemic agents. Participants were wearing light clothing during height and weight measurement. WC was measured at the umbilical level, with the participant in a standing position. WHtR was defined as WC (cm) divided by height (cm). Body mass index (BMI) was defined as weight (kg) divided by the height (m) squared. Public-health nurses obtained information on participants’ smoking, drinking, and medical histories.

### Endpoint determination

The endpoint determination has been previously reported.^14^^–^^16^ The endpoints of the present study were (1) date of first coronary heart disease (CHD) or stroke event; (2) date of death; (3) date of departure from Suita city; or (4) December 31, 2007. The first step in the survey of CHD and stroke was checking the health status of all participants by means of clinical visits every 2 years and a yearly questionnaire (by mail or telephone). For the second step, in-hospital medical records of participants suspected of having CHD or stroke were reviewed by registered hospital physicians, who were blinded to the baseline information. In addition, to complete the survey, we also conducted a systematic search of death certificates to identify cases of fatal CHD and stroke. In Japan, all death certificates are forwarded to the Ministry of Health, Welfare, and Labour and coded for the National Vital Statistics. The criteria for myocardial infarction were based on the World Health Organization Monitoring of Trends and Determinants in Cardiovascular Disease projects.^17^ In addition to myocardial infarction, we also evaluated coronary angioplasty, coronary artery bypass grafting, and sudden cardiac death, all of which were included in the definition of CHD. Stroke was defined according to criteria from the US National Survey of Stroke and was confirmed by computed tomography.^18^ Classification of stroke was based on examination of computed tomography scans, magnetic resonance images, and autopsy findings.

### Statistical analysis

To assess the association between age and WHtR, we analyzed mean WC, height, and WHtR according to age in men and women. Pearson product-moment correlation coefficients between height and waist were calculated by sex and age group (30–49, 50–69, ≥70 years). Participants were categorized based on quartiles of WHtR by sex and age group. To compare baseline characteristics among WHtR quartiles, analysis of variance was used for continuous variables and the χ^2^ test was used for dichotomous and categorical variables.

The Cox proportional hazards model was used to investigate the association between WHtR and CVD risk only among participants aged 50 to 69 years and 70 years or older, because there were too few CVD cases (men: 17, women: 11) for statistical analysis among those aged 30 to 49 years. Interaction terms were added to the models to assess the interaction between age and WHtR quartile for the risk of CVD. Hazard ratios (HRs) and 95% CIs were computed, and the lowest quartile of WHtR was defined as the reference group. To adjust for confounding factors, we included age, smoking status (current, quit, or never), and drinking status (current, quit, or never) in the model. Cardiometabolic risk factors such as hypertension, diabetes, and hypercholesterolemia were not included in the model because central obesity is upstream in the “metabolic domino”.^19^ However, in sensitivity analysis, we adjusted for hypertension, diabetes, and hypercholesterolemia to confirm that WHtR was an independent risk factor. The same analysis was performed for WC. In addition, to further assess cut-off points for WHtR, the highest quartile was dichotomized by median WHtR (ie, upper Q4 and lower Q4), and HRs and 95% CIs were estimated. The likelihood ratio test was used to compare the predictive values of WHtR with WC, as follows. First, we calculated the −2 logarithm likelihood for the model including the confounding factors, age, smoking, and drinking status (*−2 ln[L_c_]*). Second, we calculated the −2 logarithm likelihood for the model including the confounding factors plus WHtR (*−2 ln[L_c +WHtR_ ]*). The difference, ie, (*−2 ln[L_c_] − (−2 ln[L_c +WHtR_ ])*), had an approximate χ^2^ distribution with 1 degree-of-freedom. The same analysis was performed for WC.

All *P* values were 2-tailed, and a *P* value less than 0.05 was considered statistically significant. All statistical analyses were performed with SPSS (Version 20.0J; Japan IBM, Tokyo, Japan).

## RESULTS

During the follow-up period (mean, 13.0 years), 428 CVD events (184 CHD and 244 strokes) were observed. The Figure shows average WC, height, and WHtR by sex and age. WC in men increased up to age 50 years, remained almost unchanged from age 50 to 69 years, and decreased at age 70 years or older. WC in women younger than 75 years increased with advancing age and decreased in women aged 75 years or older, as compared with women aged 70 to 74 years. Height decreased with advancing age in both sexes. WHtR in men increased until approximately age 60 years. WHtR in women younger than 75 years increased with advancing age. The Pearson product-moment correlation coefficients (95% CI) between height and WC were 0.16 (0.09–0.22), 0.24 (0.19–0.30), and 0.13 (0.04–0.22) among men aged 30 to 49, 50 to 69, and 70 years or older, respectively, and 0.07 (0.01–0.13), 0.07 (0.02–0.13), 0.09 (−0.003–0.19) among women in the respective age groups.

**Figure.**
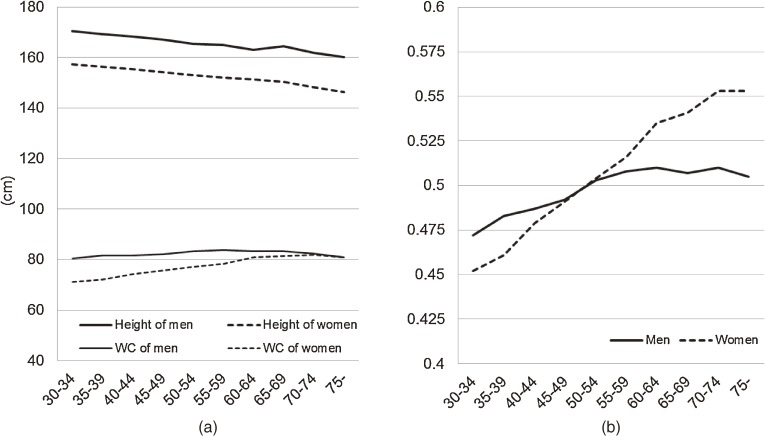
(a) Average WC (waist circumference), height, and (b) waist-to-height ratio according to age (The Suita Study, Japan)

Tables 1 and 2 summarize the baseline characteristics according to WHtR quartile (results among men and women aged 30–49 years are shown in eTable 1.) The prevalence of hypertension significantly differed by WHtR quartile, except among men aged 70 years or older. The prevalence of hypercholesterolemia and diabetes significantly differed by WHtR quartile among men and women aged 50 to 69 years.

**Table 1.** Baseline characteristics of men, according to age group and quartile of waist-to-height ratio: The Suita Study, Japan

	Q1 (low)	Q2	Q3	Q4 (high)	*P*-value
Age 50–69 years					
No. of subjects	308	304	304	308	
Waist-to-height ratio	0.374–0.475	0.476–0.508	0.509–0.536	0.537–0.761	
Waist, cm	74.0 ± 4.3	81.2 ± 2.9	85.7 ± 3.1	92.8 ± 5.5	<0.01
Height, cm	165.0 ± 5.3	164.9 ± 5.6	164.4 ± 5.4	163.7 ± 5.3	0.01
Age, years	59.0 ± 5.3	59.1 ± 5.2	59.1 ± 5.5	59.4 ± 5.3	0.77
Body mass index, kg/m^2^	20.1 ± 1.7	22.1 ± 1.5	23.7 ± 1.5	25.9 ± 2.3	<0.01
Hypertension, %	31	35	45	51	<0.01
Diabetes, %	6	7	9	11	0.045
Hypercholesterolemia, %	23	28	40	35	<0.01
Smoking status (current/quit/never), %	58/25/17	50/31/19	46/35/19	44/38/19	0.01
Drinking status (current/quit/never), %	79/2/19	74/4/22	79/4/17	76/4/21	0.58
Age ≥70 years					
No. of subjects	120	120	124	119	
Waist-to-height ratio	0.352–0.472	0.473–0.508	0.509–0.543	0.544–0.688	
Waist, cm	70.6 ± 5.0	79.8 ± 3.4	84.9 ± 3.3	92.2 ± 5.6	<0.01
Height, cm	162.5 ± 6.0	162.2 ± 5.7	161.3 ± 5.3	159.3 ± 6.0	<0.01
Age, years	74.0 ± 3.0	73.5 ± 2.7	74.1 ± 2.7	73.7 ± 2.9	0.40
Body mass index, kg/m^2^	18.5 ± 1.7	21.3 ± 1.7	22.7 ± 1.4	25.6 ± 2.0	<0.01
Hypertension, %	42	44	51	57	0.07
Diabetes, %	4	7	7	8	0.70
Hypercholesterolemia, %	23	29	26	31	0.46
Smoking status (current/quit/never), %	37/48/16	42/41/18	38/47/15	30/50/19	0.66
Drinking status (current/quit/never), %	58/8/33	62/11/28	62/6/32	65/8/28	0.73

Continuous data with a normal distribution were analyzed with analysis of variance: mean ± SD.

Dichotomous and categorical data were analyzed with the χ^2^ test.

Q, quartile; hypertension was defined as systolic blood pressure/diastolic blood pressure ≥ 140/90 mm Hg or current use of antihypertensive medications; diabetes was defined as a fasting plasma glucose level ≥ 7.0 mmol/L, a non-fasting plasma glucose level ≥ 11.1 mmol/L, or current use of antidiabetic medications; hypercholesterolemia was defined as a total serum cholesterol level ≥ 5.7 mmol/L or current use of antihyperlipidemic medications.

**Table 2.** Baseline characteristics of women, according to age group and quartile of waist-to-height ratio: The Suita Study, Japan

	Q1 (low)	Q2	Q3	Q4 (high)	*P*-value
Age 50–69 years					
No. of subjects	337	340	335	339	
Waist-to-height ratio	0.348–0.472	0.473–0.520	0.521–0.568	0.569–0.838	
Waist, cm	67.3 ± 4.1	75.4 ± 3.3	82.7 ± 3.4	92.1 ± 6.6	<0.01
Height, cm	153.0 ± 4.7	151.8 ± 4.9	152.1 ± 5.1	150.3 ± 5.2	<0.01
Age, years	57.6 ± 5.3	58.5 ± 5.3	59.5 ± 5.2	60.5 ± 5.4	<0.01
Body mass index, kg/m^2^	19.8 ± 2.0	21.7 ± 2.0	23.1 ± 2.3	25.9 ± 3.3	<0.01
Hypertension, %	21	32	36	52	<0.01
Diabetes, %	2	3	5	9	<0.01
Hypercholesterolemia, %	49	57	57	62	0.01
Smoking status (current/quit/never), %	11/2/86	11/3/86	9/3/88	12/5/84	0.43
Drinking status (current/quit/never), %	26/2/73	29/2/69	28/2/71	31/1/68	0.75
Postmenopausal, %	90	94	95	94	0.06
Age ≥70 years					
No. of subjects	103	103	103	103	
Waist-to-height ratio	0.379–0.496	0.497–0.554	0.556–0.602	0.603–0.812	
Waist, cm	68.1 ± 4.4	77.3 ± 4.1	85.6 ± 3.6	95.2 ± 6.4	<0.01
Height, cm	148.4 ± 5.5	147.7 ± 6.1	148.1 ± 5.1	145.8 ± 5.1	<0.01
Age, years	73.8 ± 2.9	73.4 ± 2.7	73.8 ± 2.7	74.0 ± 2.6	0.56
Body mass index, kg/m^2^	19.1 ± 2.1	21.3 ± 2.3	23.1 ± 2.1	26.2 ± 2.9	<0.01
Hypertension, %	53	44	50	64	0.03
Diabetes, %	2	5	6	4	0.54
Hypercholesterolemia, %	42	51	53	52	0.32
Smoking status (current/quit/never), %	12/6/83	9/4/87	6/5/89	7/5/88	0.78
Drinking status (current/quit/never), %	22/5/73	18/2/81	19/1/80	19/4/77	0.62
Postmenopausal, %	100	100	100	100	1.00

Continuous data with a normal distribution were analyzed with analysis of variance: mean ± SD.

Dichotomous and categorical data were analyzed with the χ^2^ test.

Q, quartile; hypertension was defined as systolic blood pressure/diastolic blood pressure ≥ 140/90 mm Hg or current use of antihypertensive medications; diabetes was defined as a fasting plasma glucose level ≥ 7.0 mmol/L, a non-fasting plasma glucose level ≥ 11.1 mmol/L, or current use of antidiabetic medications; hypercholesterolemia was defined as a total serum cholesterol level ≥ 5.7 mmol/L or current use of antihyperlipidemic medications.

Table 3 shows multivariable-adjusted HRs and 95% CIs for CVD and its subtypes according to WHtR quartile. A significant interaction was observed between age and WHtR for CVD among men (*P* for interaction = 0.02). Men aged 50 to 69 years in the highest quartile had significantly higher risks of CVD and CHD as compared with men in the lowest quartile; the HRs (95% CI) were 1.82 (1.13–2.92) and 2.42 (1.15–5.12), respectively. There were significant linear increases in the HRs for CVD, CHD, and ischemic stroke in men aged 50 to 69 years. After further adjustment for hypertension, diabetes, and hypercholesterolemia, the HRs (95% CI) were 1.46 (0.90–2.36) and 1.89 (0.89–4.03), respectively (eTable 3). Women aged 50 to 69 years in the highest quartile had a significantly higher risk of stroke than did those in the lowest quartile; the HR (95% CI) was 2.43 (1.01–5.85). There were significant linear increases in the HRs of CVD and stroke in women aged 50 to 69 years. After further adjustment for hypertension, diabetes, and hypercholesterolemia, the HR (95% CIs) was 2.06 (0.84–5.04) (eTable 3).

**Table 3.** Multivariable-adjusted hazard ratios for cardiovascular disease according to sex, age group, and quartile of WHtR: The Suita Study, Japan

	Q1 (low)	Q2	Q3	Q4 (high)	*P* for trend
Men					
Age 50–69 years					
Person-years	4070	3069	3879	3842	
CVD, no. of cases	28	31	32	47	
HRs	1	1.14 (0.68–1.90)	1.23 (0.74–2.05)	1.82 (1.13–2.92)	0.01
CHD, no. of cases	10	16	16	23	
HRs	1	1.57 (0.71–3.47)	1.72 (0.77–3.80)	2.42 (1.15–5.12)	0.02
Stroke, no. of cases	18	15	16	24	
HRs	1	0.91 (0.46–1.81)	0.95 (0.48–1.87)	1.56 (0.84–2.89)	0.16
Ischemic stroke, no. of cases	10	9	15	18	
HRs	1	0.99 (0.40–2.43)	1.59 (0.71–3.56)	2.06 (0.94–4.49)	0.04
Age ≥70 years					
Person-years	1055	1128	1193	1155	
CVD, no. of cases	21	29	27	30	
HRs	1	1.36 (0.77–2.39)	1.09 (0.62–1.93)	1.36 (0.78–2.38)	0.45
CHD, no. of cases	13	11	10	15	
HRs	1	0.87 (0.39–1.97)	0.63 (0.28–1.45)	1.09 (0.52–2.30)	0.99
Stroke, no. of cases	8	18	17	15	
HRs	1	2.09 (0.90–4.81)	1.79 (0.77–4.15)	1.84 (0.78–4.35)	0.29
Ischemic stroke, no. of cases	4	12	10	11	
HRs	1	2.84 (0.91–8.83)	2.22 (0.69–7.07)	2.71 (0.86–8.53)	0.18
Women					
Age 50–69 years					
Person-years	4811	4863	4477	4470	
CVD, no. of cases	16	18	21	33	
HRs	1	1.09 (0.56–2.14)	1.32 (0.69–2.54)	1.80 (0.98–3.32)	0.04
CHD, no. of cases	9	4	4	13	
HRs	1	0.47 (0.14–1.51)	0.47 (0.14–1.54)	1.35 (0.56–3.22)	0.43
Stroke, no. of cases	7	14	17	20	
HRs	1	1.85 (0.75–4.60)	2.35 (0.97–5.70)	2.43 (1.01–5.85)	0.04
Ischemic stroke, no. of cases	3	7	9	10	
HRs	1	2.09 (0.54–8.10)	2.78 (0.75–10.33)	2.35 (0.63–8.77)	0.22
Age ≥70 years					
Person-years	1095	1259	1164	1094	
CVD, no. of cases	15	15	13	24	
HRs	1	1.00 (0.48–2.08)	0.91 (0.43–1.93)	1.83 (0.95–3.53)	0.08
CHD, no. of cases	6	7	5	9	
HRs	1	1.23 (0.40–3.77)	0.98 (0.29–3.32)	1.78 (0.62–5.14)	0.34
Stroke, no. of cases	9	8	8	15	
HRs	1	0.85 (0.32–2.23)	0.88 (0.34–2.29)	1.92 (0.83–4.45)	0.11
Ischemic stroke, no. of cases	5	4	4	9	
HRs	1	0.83 (0.22–3.16)	0.77 (0.21–2.91)	1.99 (0.66–6.04)	0.21

Multivariable adjustment was performed for age, smoking, and drinking status. Parentheses indicate 95% CIs for HRs.

Abbreviations: WHtR, waist-to-height ratio; Q, quartile; CVD, cardiovascular disease; CHD, coronary heart disease; HR, hazard ratio.

When men aged 50 to 69 years in the highest quartile were dichotomized by median WHtR (0.56), the HR (95% CI) for CVD was 1.37 (0.76–2.46) for those in the lower WHtR group and 2.34 (1.38–3.97) for those in the upper WHtR group (eTable 2). When women aged 70 years or older in the highest quartile were dichotomized by median WHtR (0.65), the HR for CVD was 1.42 (0.63–3.18) for those in the lower WHtR group and 2.33 (1.10–4.94) for those in the upper WHtR group. After adjustment for hypertension, diabetes, and hypercholesterolemia, the HRs in the upper WHtR decreased but remained significant, ie, 1.78 (1.04–3.05) among men aged 50 to 69 years and 2.16 (1.02–4.61) among women aged 70 years or older.

Table 4 shows the HRs and 95% CIs for CVD in relation to WC quartile. Among men aged 50 to 69 years in the highest quartile, the HR for CVD was 1.63 (1.03–2.59), although the HRs of CVD did not show a significant linear increase in this group. Among women aged 50 to 69 years, a significant linear increase was observed in the HRs for CVD (*P* for trend = 0.04). However, after further adjustment for hypertension, diabetes, and hypercholesterolemia, these associations were no longer significant among men or women.

**Table 4.** Multivariable-adjusted hazard ratios for cardiovascular disease according to sex, age group, and quartile of WC: The Suita Study, Japan

	Q1 (low)	Q2	Q3	Q4 (high)	*P* for trend
Men					
Age 50–69 years					
Person-years	4078	4004	3872	3806	
CVD, no. of cases	32	33	29	44	
HRs	1	1.07 (0.66–1.75)	0.97 (0.58–1.61)	1.63 (1.03–2.59)	0.06
CHD, no. of cases	13	17	12	23	
HRs	1	1.28 (0.62–2.63)	0.96 (0.44–2.12)	2.02 (1.02–4.02)	0.07
Stroke, no. of cases	19	16	17	21	
HRs	1	0.97 (0.50–1.88)	0.96 (0.49–1.86)	1.43 (0.76–2.67)	0.31
Ischemic stroke, no. of cases	13	9	13	17	
HRs	1	0.80 (0.34–1.87)	1.07 (0.49–2.31)	1.64 (0.79–3.41)	0.15
Age ≥70 years					
Person-years	999	1208	1200	1124	
CVD, no. of cases	25	28	27	27	
HRs	1	0.94 (0.55–1.62)	0.91 (0.53–1.58)	1.06 (0.61–1.84)	0.87
CHD, no. of cases	14	11	12	12	
HRs	1	0.67 (0.30–1.47)	0.65 (0.30–1.43)	0.82 (0.38–1.78)	0.60
Stroke, no. of cases	11	17	15	15	
HRs	1	1.29 (0.60–2.77)	1.21 (0.55–2.66)	1.36 (0.62–2.99)	0.52
Ischemic stroke, no. of cases	5	10	10	12	
HRs	1	1.70 (0.58–4.98)	1.82 (0.62–5.37)	2.26 (0.79–6.47)	0.14
Women					
Age 50–69 years					
Person-years	4669	4685	5046	4221	
CVD, no. of cases	15	18	25	30	
HRs	1	1.19 (0.60–2.36)	1.43 (0.75–2.71)	1.87 (1.00–3.51)	0.04
CHD, no. of cases	7	5	5	13	
HRs	1	0.74 (0.24–2.34)	0.65 (0.21–2.08)	1.86 (0.73–4.72)	0.18
Stroke, no. of cases	8	13	20	17	
HRs	1	1.56 (0.65–3.77)	2.06 (0.90–4.70)	1.93 (0.82–4.54)	0.11
Ischemic stroke, no. of cases	4	6	9	10	
HRs	1	1.44 (0.41–5.10)	1.70 (0.52–5.54)	2.00 (0.62–6.52)	0.23
Age ≥70 years					
Person-years	1175	1234	1046	1157	
CVD, no. of cases	16	16	15	20	
HRs	1	1.05 (0.52–2.11)	1.11 (0.54–2.25)	1.45 (0.74–2.83)	0.28
CHD, no. of cases	8	6	7	6	
HRs	1	0.85 (0.29–2.49)	1.21 (0.43–3.43)	0.88 (0.30–2.59)	0.98
Stroke, no. of cases	8	10	8	14	
HRs	1	1.24 (0.49–3.14)	1.10 (0.41–2.93)	2.00 (0.83–4.87)	0.15
Ischemic stroke, no. of cases	5	4	4	9	
HRs	1	0.85 (0.23–3.21)	0.93 (0.25–3.47)	1.86 (0.61–5.61)	0.24

Multivariable adjustment was performed for age, smoking, and drinking status. Parentheses indicate 95% CIs for HRs.

Abbreviations: WC, waist circumference; Q, quartile; CVD, cardiovascular disease; CHD, coronary heart disease; HR, hazard ratio.

The χ^2^ values for the likelihood ratio test were 6.49 (*P* = 0.01) for WHtR and 3.63 (*P* = 0.06) for WC among men aged 50 to 69 years, and 4.45 (*P* = 0.03) for WHtR and 4.54 (*P* = 0.03) for WC among women aged 50 to 69 years.

## DISCUSSION

Our main findings were that WHtR was significantly positively associated with CVD and CHD risk among men aged 50 to 69 years and with stroke risk among women aged 50 to 69 years. Among men, there was a significant interaction between age and WHtR for CVD incidence. Among women aged 50 to 69 years, there was a borderline association between a WHtR in the highest quartile and increased CVD risk. In addition, among women aged 70 years or older, a WHtR in the upper level of the highest quartile was associated with significantly elevated CVD risk. These findings suggest that the association between WHtR and CVD incidence differs according to age and sex.

Two previous studies, in the United States and China, reported that the association between WHtR and CVD risk was stronger among younger adults as compared with elderly adults.^12^^,^^13^ We too observed a significantly stronger association between WHtR and CVD risk among relatively young adults (age 50–69 years) as compared with elderly adults (age ≥70 years), which supports the results of previous studies. Consequently, these findings suggest that age stratification is important in estimating the association between WHtR and CVD risk.

In this population, physical frame, eg, WC and height, differed by age group. It has been reported that WC and the ratio of abdominal fat to whole-body fat differ by age.^9^^,^^10^ In addition, the National Health and Nutrition Examination Survey in Japan noted that height clearly differed by generation.^11^ This generational difference in physical frame, as well as aging, could lead to age differences in the association between WHtR and CVD risk.

A recent meta-analysis reported an optimal cut-off point of 0.50 for WHtR in both sexes.^7^ However, the present findings suggest that, regardless of age or sex, a cut-off of 0.50 is somewhat low for identifying individuals at higher risk for CVD. The association with CVD risk was of at least borderline significance for a WHtR in the fourth quartile, except among men aged 70 years or older. Additional analyses showed that the risks markedly increased, particularly in the upper level of the fourth WHtR quartile, among men aged 50 to 69 years and women aged 70 years and older. These results suggest the presence of a threshold rather than a dose-response relation for WHtR, although the present sample was too small to confirm this hypothesis. Additionally, we think that cut-offs should be set in relation to age and sex. On the basis of our results, we propose the following cut-offs (which do not include men aged 70 years or older): 0.560 for men aged 50 to 69 years, 0.569 for women aged 50 to 69 years, and 0.647 for women aged 70 years or older.

The risk of CVD among men aged 50 to 69 years, and women aged 70 years, in the upper level of the highest quartile was significantly elevated even after adjustment for hypertension, hyperlipidemia, and diabetes. We believe that there are 2 possible explanations for this finding. First, an extremely high WHtR might actually be an independent risk factor ie, separate from classical cardiometabolic risks. It has been reported that abdominal obesity is related to increased levels of plasminogen activator inhibitor-1, which can lead to blood coagulation.^20^ Such background mechanisms might be important. Second, our findings could be due to insufficient adjustment for confounders in the Cox regression model. Irrespective of the reason, men aged 50 to 69 years, and women aged 70 years or older, with extremely high WHtRs have a considerably higher risk for CVD and should be closely monitored.

We previously investigated the association between WC and CVD risk without age stratification^21^ and found a significant association between WC and the risks of CVD and stroke among women but no significant association among men. However, the present age-stratified analysis of WC suggests that our previous results were substantially influenced by age. Therefore, we compared WHtR and WC in relation to CVD in analysis stratified by age group and found that the HRs associated with the highest quartile of WHtR were higher than those associated with WC among middle-aged men and that the predictive value of WHtR was greater than that of WC. Several previous studies reported similar results^12^^,^^22^^–^^24^; therefore our findings are consistent with those of previous studies. In contrast, WHtR and WC had similar predictive values for CVD among women in the present study. Many previous studies found that WHtR was similar to WC in predicting CVD risk among women.^12^^,^^22^^,^^24^^–^^26^ The effect of dividing WC by height might be limited because the correlation of WC with height is weaker among women than among men. Consequently, we believe that WHtR is a better predictor than WC, particularly among middle-aged men.

The superiority of WHtR might be explained by the fact that WHtR, as measured by computed tomography, was more closely correlated than WC with intra-abdominal fat,^27^ and a previous study reported that intra-abdominal fat was positively associated with number of cardiometabolic risk factors.^28^ In addition, shorter adults tend to have more cardiometabolic risk factors than do taller individuals with a similar WC.^29^ This suggests that WHtR, ie, dividing WC by height, is more strongly related than WC to cardiometabolic risk factors. Thus, we believe that WHtR better reflects the accumulation of cardiometabolic risks and leads to superior prediction of CVD.

BMI, along with indices of central obesity, has been an important obesity index in predicting CVD incidence,^30^ although a meta-analysis reported that the predictive power of WHtR for CVD was higher than that of BMI.^7^ Another report found a significant association between BMI and CVD after adjustment for WHtR^12^ and suggested that WHtR and BMI are independently associated with CVD risk. Therefore, it might be better to use both BMI and WHtR to assess obesity.

Our study has several limitations. First, the number of cases of CVD among participants aged 30 to 49 years was insufficient for statistical analysis. Further study is required to confirm an association between WHtR and CVD risk among younger adults. Second, the effect of visceral fat could not be estimated because we did not use computed tomography to measure abdominal fat distribution. Third, changes in WHtR during the follow-up period were not considered in the present study. Finally, because WC was measured once, the estimated risks might have been underestimated because of regression dilution bias.^31^

In conclusion, the present findings suggest that WHtR is useful in identifying middle-aged Japanese at higher risk of CVD and is more predictable than WC in determining CVD risk, especially among men. In addition, the data indicate that WHtR cut-off points should be set according to sex and age. This study enrolled a limited Japanese population, and further studies with larger and more ethnically diverse samples are required to confirm our findings.

## ONLINE ONLY MATERIALS

eTable 1. Baseline characteristics and CVD incidence among men and women aged 30–49 years according to quartile of waist-to-height ratio: the Suita Study, Japan.

eTable 2. Multivariable-adjusted hazard ratios for cardiovascular disease in the upper and lower fourth quartile of WHtR according to sex and age group: the Suita Study, Japan.

eTable 3. Multivariable-adjusted hazard ratios for cardiovascular disease according to sex, age group, and quartile of WHtR: the Suita Study, Japan.

Abstract in Japanese.
