# Supplementary material for: Effect of Age on the Association Between Waist-to-Height Ratio and Incidence of Cardiovascular Disease: The Suita Study
Source: J Epidemiol. 2013 Sep 5;23(5):351–9. doi: 10.2188/jea.JE20130004 (PMC3775529; doi:10.2188/jea.JE20130004)
Supplement: eTable 1. — Baseline characteristics and CVD incidence among men and women aged 30–49 years according to quartile of waist-to-height ratio: the Suita Study, Japan. [file je-23-351-s001.pdf]

eTable 1. Baseline characteristics and CVD incidence among men and women aged 30-49 years according to quartile of waist-to-height ratio: the Suita Study, Japan

	Q1 (low)	Q2	Q3	Q4 (high)	P
Men aged 30-49 years					
No. of subjects	222	225	222	224	
Baseline character					
Waist-to-height ratio	0.374-0.452	0.453-0.481	0.482-0.514	0.515-0.650	
Waist, cm	73.0±3.70	79.3±2.71	84.0±3.3	90.3±5.4	<0.01
Height, cm	170.2±5.2	169.2±5.3	168.7±5.7	166.7±5.5	<0.01
Age, years	39.0±5.5	39.4±5.4	40.6±5.2	41.6±5.3	<0.01
Body mass index, kg/m^2	20.1±1.6	22.0±1.4	23.8±1.6	26.2±2.4	<0.01
Hypertension, %	6	15	17	29	<0.01
Diabetes, %	3	1	1	5	0.11
Hypercholesterolemia, %	11	20	28	34	<0.01
Smoking status (current/quit/never), %	71/13/16	56/18/26	59/22/20	56/20/24	0.01
Drinking status (current/quit/never), %	76/1/23	84/1/15	85/1/14	80/2/18	0.24
Incidence of any CVD, n,	5	4	2	6	
Women aged 30-49 years					
No. of subjects	282	281	283	279	
Baseline character					
Waist-to-height ratio	0.352-0.431	0.432-0.467	0.468-0.506	0.507-0.778	
Waist, cm	64.1±3.3	70.0±2.9	75.3±2.9	85.1±6.7	<0.01
Height, cm	157.4±5.1	155.9±4.9	155.0±4.8	154.5±5.2	<0.01
Age, years	38.6±4.9	40.1±5.3	41.1±5.3	42.5±4.9	<0.01
Body mass index, kg/m^2	19.3±1.6	20.8±2.0	21.8±2.1	24.6±3.2	<0.01
Hypertension, %	2	6	7	17	<0.01
Diabetes, %	1	0	1	4	<0.01
Hypercholesterolemia, %	13	21	17	23	0.01
Smoking status (current/quit/never), %	16/2/82	13/6/82	17/1/82	16/6/78	<0.01
Drinking status (current/quit/never), %	44/1/55	43/3/54	45/1/54	40/0/60	0.13
Postmenopausal, %	4	7	8	13	<0.01
Incidence of any CVD, n,	3	1	2	5	

Continuous data with a normal distribution were analyzed with analysis of variance: mean ± SD.

Dichotomous and categorical data were analyzed with the χ^2 test.

Q, quartile; hypertension was defined as systolic blood pressure/diastolic blood pressure ≥ 140/90 mm Hg or current use of antihypertensive medications; diabetes was defined as a fasting plasma glucose level ≥ 7.0 mmol/L, a non-fasting plasma glucose level ≥ 11.1 mmol/L, or current use of antidiabetic medications; hypercholesterolemia was defined as a total serum cholesterol level ≥ 5.7 mmol/L or current use of antihyperlipidemic medications.
