# Supplementary material for: Effect of Age on the Association Between Waist-to-Height Ratio and Incidence of Cardiovascular Disease: The Suita Study
Source: J Epidemiol. 2013 Sep 5;23(5):351–9. doi: 10.2188/jea.JE20130004 (PMC3775529; doi:10.2188/jea.JE20130004)
Supplement: eTable 2. — Multivariable-adjusted hazard ratios for cardiovascular disease in the upper and lower fourth quartile of WHtR according to sex and age group: the Suita Study, Japan. [file je-23-351-s002.pdf]

eTable 2. Multivariable-adjusted hazard ratios for cardiovascular disease in the upper and lower fourth quartile of WHtR according to sex and age group: the Suita Study, Japan

	Q1	Lower Q4	Upper Q4
**Men**			
Age 50-69 years			
WHtR		0.537-0.559	0.560-0.761
No. of cases	28	19	28
Model 1	1	1.37 (0.76-2.46)	2.34 (1.38-3.97)
Model 2	1	1.15 (0.63-2.09)	1.78 (1.04-3.05)
Age ≥70 years			
WHtR		0.544-0.570	0.571-0.688
No. of cases	21	16	14
Model 1	1	1.71 (0.89-3.30)	1.10 (0.56-2.18)
Model 2	1	1.63 (0.84-3.17)	0.99 (0.50-1.95)
**Women**			
Age 50-69 years			
WHtR		0.569-0.600	0.601-0.838
No. of cases	16	18	15
Model 1	1	1.97 (0.99-3.91)	1.63 (0.80-3.34)
Model 2	1	1.66 (0.83-3.31)	1.20 (0.57-2.52)
Age ≥70 years			
WHtR		0.603-0.646	0.647-0.812
No. of cases	15	10	14
Model 1	1	1.42 (0.63-3.18)	2.33 (1.10-4.94)
Model 2	1	1.41 (0.63-3.16)	2.16 (1.02-4.61)

The highest (4th) quartile of WHtR was dichotomized by using the median WHtR in the fourth quartile (upper Q4 and lower Q4), and hazard ratios with 95% CIs for cardiovascular disease were estimated with the lowest (1st) quartile (Q1) as reference.

Model 1 adjusted for age, smoking, and drinking status. Model 2 adjusted for the variables in model 1 plus hypertension, diabetes, and hypercholesterolemia.

Parentheses indicate 95% CIs of hazard ratios.

Abbreviations: WHtR, waist-to-height ratio; Q, quartile.
