# Supplementary material for: Effect of Age on the Association Between Waist-to-Height Ratio and Incidence of Cardiovascular Disease: The Suita Study
Source: J Epidemiol. 2013 Sep 5;23(5):351–9. doi: 10.2188/jea.JE20130004 (PMC3775529; doi:10.2188/jea.JE20130004)
Supplement: eTable 3. — Multivariable-adjusted hazard ratios for cardiovascular disease according to sex, age group, and quartile of WHtR: the Suita Study, Japan. [file je-23-351-s003.pdf]

eTable 3. Multivariable-adjusted hazard ratios for cardiovascular disease according to sex, age group, and quartile of WHtR: the Suita Study, Japan

	Q1 (low)	Q2	Q3	Q4 (high)	P for trend
Men aged 50-69 years					
CVD	1	1.06 (0.63-1.77)	1.03 (0.61-1.73)	1.46 (0.90-2.36)	0.12
CHD	1	1.48 (0.67-3.28)	1.35 (0.60-3.03)	1.89 (0.89-4.03)	0.13
Stroke	1	0.82 (0.41-1.64)	0.83 (0.42-1.65)	1.29 (0.68-2.42)	0.41
Ischemic stroke	1	0.86 (0.35-2.12)	1.32 (0.58-3.01)	1.59 (0.72-3.54)	0.14
Men aged ≥70 years					
CVD	1	1.34 (0.76-2.36)	1.04 (0.59-1.84)	1.25 (0.71-2.19)	0.68
CHD	1	0.86 (0.38-1.95)	0.61 (0.26-1.39)	0.99 (0.47-2.11)	0.82
Stroke	1	2.07 (0.90-4.79)	1.71 (0.73-3.97)	1.68 (0.71-3.99)	0.42
Ischemic stroke	1	2.84 (0.91-8.87)	2.07 (0.65-6.64)	2.41 (0.76-7.62)	0.30
Women aged 50-69 years					
CVD	1	1.01 (0.51-1.99)	1.15 (0.59-2.23)	1.43 (0.76-2.68)	0.21
CHD	1	0.41 (0.12-1.33)	0.40 (0.12-1.31)	0.94 (0.38-2.34)	0.93
Stroke	1	1.78 (0.71-4.42)	2.13 (0.87-5.18)	2.06 (0.84-5.04)	0.13
Ischemic stroke	1	1.98 (0.51-7.73)	2.48 (0.66-9.27)	1.99 (0.53-7.56)	0.37
Women aged ≥70 years					
CVD	1	1.06 (0.51-2.21)	0.92 (0.43-1.96)	1.75 (0.91-3.39)	0.12
CHD	1	1.24 (0.40-3.82)	0.89 (0.26-3.03)	1.60 (0.55-4.63)	0.49
Stroke	1	0.89 (0.34-2.37)	0.91 (0.35-2.39)	1.90 (0.82-4.41)	0.13
Ischemic stroke	1	0.87 (0.22-3.37)	0.77 (0.20-2.92)	1.98 (0.65-6.03)	0.23

Multivariable adjustments were performed for age, smoking, drinking status, diabetes, hypertension and hypercholesterolemia. Parentheses indicate 95% CIs of HRs.

Abbreviations: WHtR, waist-to-height ratio; Q, quartile; CVD, cardiovascular disease; CHD, coronary heart disease.
