# Supplementary material for: Effect of Age on the Association Between Waist-to-Height Ratio and Incidence of Cardiovascular Disease: The Suita Study
Source: J Epidemiol. 2013 Sep 5;23(5):351–9. doi: 10.2188/jea.JE20130004 (PMC3775529; doi:10.2188/jea.JE20130004)
Supplement: Abstract in Japanese. [file je-23-351-s004.pdf]

腹囲・身長比と循環器疾患発症の関連における年齢の影響：吹田研究

辰巳友佳子[1,2]、渡邉至[1]、小久保喜弘[1]、西村邦宏[1]、東山綾[3]、岡村智教[4]、岡山明[5]、宮本恵宏[1]
1, 国立循環器病研究センター
2, 大阪大学大学院医学系研究科　総合ヘルスプロモーション科学
3, 兵庫医科大学　環境予防医学
4, 慶應義塾大学医学部 衛生学公衆衛生学
5, 公益財団法人結核予防会　第一健康相談所総合健診センター

背景：腹囲だけでなく、腹囲・身長比も循環器疾患およびメタボリックシンドロームのスクリーニングに有用であることが報告されているが、年齢の影響を考慮した報告は限られている。そこで、本研究では腹囲・身長比と循環器疾患の発症の関連を年齢階級別に検討した。
方法：1989-1994 年に大阪府吹田市住民から無作為抽出された循環器疾患（冠動脈疾患・脳卒中）の既往のない 30-83 歳の 5488 名（女性 2888 名、男性 2600 名）を平均 13 年間追跡した。性別・年齢階級別(50-69 歳、70 歳以上)に腹囲・身長比の四分位と循環器疾患発症との関連を、Cox 比例ハザードモデルを用いて検討した。
結果：50-69歳男性において、総循環器疾患および冠動脈疾患で有意にリスクが上昇し、第4四分位のハザード比(95%信頼区間)は、第1四分位を基準として、それぞれ1.82（1.13-2.92）、2.42(1.15-5.12)であった。女性では脳卒中で有意にリスクが上昇し、ハザード比は2.43（1.01-5.85)であった。男女とも、70歳以上では有意なリスクの上昇は見られなかった。尤度比検定より、腹囲・身長比は50-69歳の男性において腹囲より高い予測力を示した。
結論：腹囲・身長比と循環器疾患発症の関係は年齢階級により異なっていた。腹囲・身長比は日本人の中年男女において循環器疾患発症のハイリスク者を特定するのに有用であり、特に中年男性においては、腹囲よりも予測力が高かった。

キーワード：腹囲・身長比、年齢、循環器疾患、コホート研究
